# Social Distress among Cancer Patients: Differential Effects of Risk Factors and Attenuating Role of Culturally Specific Social Support

**DOI:** 10.3390/healthcare11131876

**Published:** 2023-06-28

**Authors:** Omar B. Da’ar, Hoda Jradi, Mohammad Alkaiyat, Ashwaq Alolayan, Abdul Rahman Jazieh

**Affiliations:** 1 Department of Health Systems Management, College of Public Health and Health Informatics, King Saud bin Abdulaziz University for Health Sciences, Riyadh 11481, Saudi Arabia; 2 King Abdullah International Medical Research Center, Riyadh 11481, Saudi Arabia; hoda.jradi@gmail.com (H.J.); alkaiyatmo@gmail.com (M.A.); ashwaqonco@gmail.com (A.A.); 3 Department of Community and Environmental Health, College of Public Health and Health Informatics, King Saud bin Abdulaziz University for Health Sciences, Riyadh 11481, Saudi Arabia; 4 Department of Oncology, King Abdulaziz Medical City, Riyadh 11426, Saudi Arabia; 5 Cincinnati Cancer Advisors, Cincinnati, OH 45212, USA; jaziehoncology@gmail.com

**Keywords:** cancer patients, social distress, social support, bounded logistic quantile regression

## Abstract

**Introduction**: We investigated the association between social distress or toxicity and patients’ clinical conditions, demographic characteristics, and social support and networks, and whether this association differs along the distribution of patients’ distress levels. This study included 156 patients treated at King Abdulaziz Medical City, Riyadh, Saudi Arabia. **Methods**: We used the previously validated Social Toxicity Assessment Tool in Cancer (STAT-C) to assess cancer patients’ distress. We analyzed distress level, the outcome variable of interest, and covariates to show distribution and identify associations. We then used logistic quantile regression for bounded outcomes to assess the association between social distress or toxicity and patients’ clinical conditions, demographic characteristics, and social support and network. As an extension, we examined the interaction between disease status and social support, focusing on the moderating role of social support in attenuating the impact of disease status on social distress. **Results**: The median age of the patients was 51.2 (SD = 21.4, range 22 to 89), with 48.1% being older than 50 years. Of the 156 cancer patients analyzed, 82 (52.6%) were classified as burdened, and 50% of those with uncontrolled disease status were socially distressed. However, there were more socially distressed patients diagnosed within a year and patients undergoing treatment. There was a greater number of patients who shared their diagnosis with family, colleagues, and neighbors with social distress. The odds of suffering from social distress were higher in younger patients (50 years or younger) than in older patients. Social distress was lower in patients who underwent combined chemotherapy, surgery, and radiation compared with patients who received a single treatment regimen (OR = 0.65, CI, −0.820 to −0.036, *p* = 0.033). The odds of social distress were 67% higher in patients diagnosed within one year than in patients diagnosed more than one year prior (OR = 1.664, CI, 0.100–0.918, *p* = 0.015). Patients with uncontrolled disease conditions who shared their diagnosis and treatment with social networks were 48% less likely to experience social distress. Thus, sharing cancer diagnoses with social networks has a statistically significant moderating effect by attenuating the impact of disease status on social distress. **Conclusion**: Understanding the risk factors for social distress may be important for cancer management. Additionally, identifying the moderating role that patients’ sharing of cancer diagnoses in social networks plays in attenuating the impact of disease status on social distress may provide healthcare providers with valuable insights for holistic culture-specific care.

## 1. Introduction

Without social support and networks, conventional cancer interventions or therapies may be less than optimal in improving clinical outcomes. Support, as an intervention strategy, assists patients in adjusting to stressful conditions [1]. Patients who do not have appropriate support from a partner or family members appear to be more vulnerable [2]. While the well-known stress-buffering hypothesis literature posits that the perceived availability of social support reduces the negative relationship between perceived chronic condition-related stress and health and quality of life [3,4] there is limited evidence on these relationships in the context of cancer patients in Saudi Arabia, a country where evidence shows cancer is a sensitive topic and most older patients do not share and discuss their conditions [5]. An interesting important question is whether sharing diagnosis and treatment with a culturally specific social network has a moderating effect by attenuating the impact of disease status (e.g., uncontrolled disease conditions) on social distress.

Socioeconomic problems affect the well-being of cancer patients [6,7,8]. Social aspects are at the heart of their well-being and the conditions in which they live [7,9]. Negative social and economic conditions can lead to poorer subjective well-being of patients and distress and affect quality of life (QoL) [7]. QoL has been considered as a component of individuals’ sensitivities to their situation in life in culture-specific and value systems where they stay relative to their aspirations, expectations, principles, and concerns [10]. Social distress or toxicity is the extent to which social relationships, social activities, and financial problems experienced by cancer patients may impede both their coping mechanisms and their response to diagnosis and treatment [11]. A cancer diagnosis is associated with a negative impact on various aspects of a patient’s life, including social and financial aspects [8,10,12,13,14]. Cancer diagnosis and treatment present challenges to patients in numerous areas of social life, including family life, relationships with caregivers, work, income, leisure activities, and relationships with healthcare providers [15,16,17,18,19]. Cancer treatments can be lengthy and place significant demands on patients and their families in terms of regular schedules, financial obligations, and daily needs [20,21].

There are several risk factors for persistent distress in newly diagnosed cancer patients. Evidence shows that females, younger patients, and patients receiving combined therapies such as chemotherapy and radiation are more vulnerable to higher distress [22]. However, social support has long been considered an important factor in cancer, even though patients are often isolated and lack support because of the stigma associated with the disease [23]. Evidence suggests that social networks and social support mechanisms are associated with higher QoL after a cancer diagnosis [24]. Evidence also suggests that patients who share information about their experience with cancer can improve their quality of life and that attachment security appears to promote social sharing [25]. The lack of close ties and perceived sources of emotional support has been associated with increased cancer death rates, particularly in patients who lacked close friends and relatives [26]. Less isolated patients and patients receiving various types of social support including compassion have been associated with higher QoL measures for breast cancer survivors [24,27].

The well-known stress-buffering hypothesis states that the perceived availability of social support eliminates or attenuates the negative relationship between perceived chronic condition-related stress and health and QoL [3,4]. While there have been studies of cancer patient outcomes in Saudi Arabia, particularly with regard to predictors of cancer patient QoL [5,28,29,30], there is limited evidence of a broader association between social distress and social networks and how support can mitigate the negative effects of cancer diagnosis and treatment.

It is critical to evaluate the impact and interactions of the determinants of social distress/toxicity in cancer patients to achieve a deeper understanding and better management in the provision of holistic patient-centered care. Such care is expected to influence the QoL of cancer patients by revealing the sensitivities of their life situation in a culture-specific value system in which they stay relative to their aspirations, expectations, principles, and concerns [10,12]. Therefore, this study examined not only how patients’ clinical conditions and characteristics affect lived experiences related to cancer-related social distress, but also how sharing diagnosis and treatment with social networks can mitigate the impact of disease status on that burden. The study also examined whether these relationships differed along the distribution of patients’ distress.

## 2. Method

### 2.1. Study Design

A cross-sectional study that included patients diagnosed with cancer, cancer patients undergoing treatment, and cancer survivors was conducted at the Oncology Department of King Abdulaziz Medical City in Riyadh, Saudi Arabia. The Institutional Review Board at King Abdullah International Medical Research Center (KAIMRC), Riyadh, Saudi Arabia, ethically approved this study.

### 2.2. Inclusion Criteria

With the help of their providers and caregivers, 156 adult patients (18 years and older) who had been diagnosed with cancer, were undergoing treatment, or had survived the disease and were being treated in the department were interviewed in January 2021. Patients agreed to participate in the study by themselves or through their caregivers and completed the social distress survey.

### 2.3. Tool Description

The study used the previously described Social Toxicity Assessment Tool in Cancer (STAT-C) [11] to assess cancer patients’ distress. The tool measures three domains: (a) the social relationship domain (eight questions) related to relationships with parents, spouse, children, siblings, friends, community members, and caregivers; (b) the social activities domain (two questions) which measures participation in social events and leisure activities; and (c) the economic impact domain (three questions). Other data collected from patients included demographic and clinical data such as age, gender, cancer type, disease stage and status, date of diagnosis, treatment received and status, and social networks, including sharing disease with family, friends, and other neighbors.

### 2.4. Data and Statistical Analysis

Data were extracted and analyzed using STATA statistical software version 16 (College Station, TX, USA). Frequencies and percentages were calculated for categorical variables and means and/or medians and standard deviations (SDs) were calculated for continuous variables. The primary outcome of interest was cancer patients’ distress level using the STAT-C tool, which consists of 14 items divided into three domains—social relationships, social activities, and economic impact. We generated the score for social distress from STAT-C, which is a bounded measure of outcome (range −28 to +28). Based on the total possible score each patient could receive for all items, we dichotomized the level of distress into socially distressed and not distressed and used the mean as a cutoff point. This was also consistent with the density plot, where most distress scores were concentrated in the interval. Because the items were reverse coded, a score below the mean was defined as socially distressed, and a score above the mean was defined as not socially distressed. We then examined the association between social distress or toxicity and patients’ clinical conditions, demographic characteristics, and social support and networks and whether this association differed along the distribution of patients’ distress levels. We also examined how social support and networks moderate the effects of disease status. Thus, we estimated the odds ratios and their corresponding 95% confidence intervals (95% CIs) of these associations using both logistic regression and bounded logistic regression analyses. Thus, we used logistic quantile regression to estimate the association between social distress and covariates, including demographic characteristics, clinical conditions, and social support and networks of cancer patients. Evidence shows the traditional statistical and classical nonparametric methods may not be suitable when the outcome variable of interest is continuous and takes values within a known range [31]. Logistic quantile regression is a method suitable for the analysis of continuous and bounded outcome variables, and it is recommended in the biomedical and epidemiological literature [31]. We also analyzed an interaction model in which we hypothesized whether patients’ sharing diagnosis and treatment with social networks moderates the effect of the disease status on social distress.

## 3. Results

**Figure 1:**
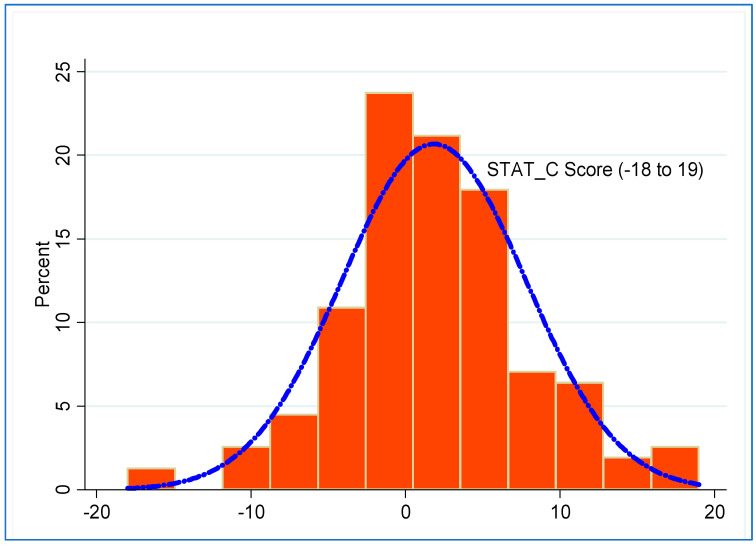
Distribution of distress score, percent (%); n = 156.

Figure 1 shows a histogram of the distribution of the STAT-C scores of the patients. The density plot shown displays where the values are concentrated over the interval. As shown in Table 1, the overall mean distress score level from the STAT-C tool was 1.84 (SD = 5.95; range −18 to 19); the socially distressed patients had a mean distress score of −2.43 (SD = 3.74), while patients no social distress had a mean score of 6.57 (SD = 4.07). Of the 156 patients, 82 (52.6%) were classified as socially distressed.

**Figure 2:**
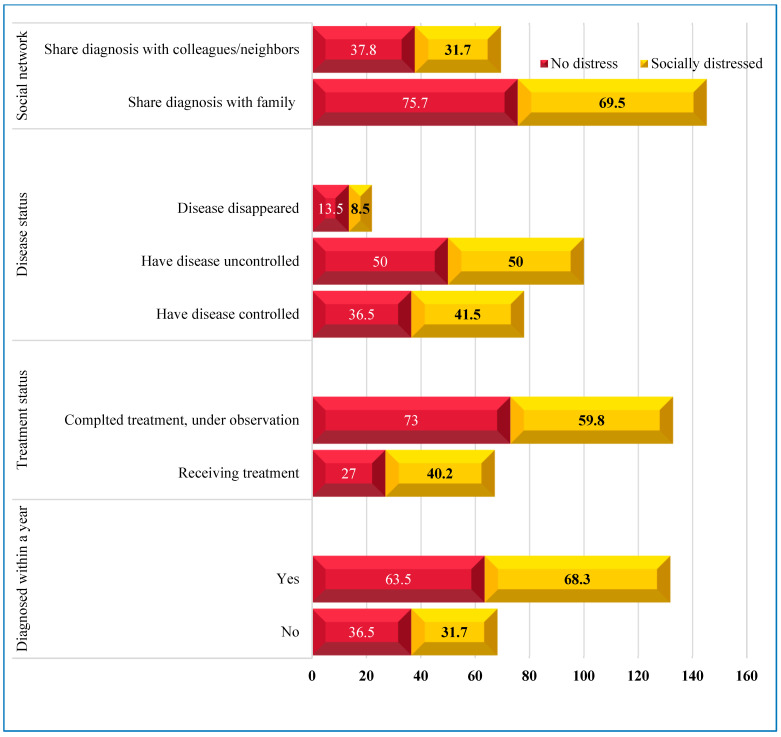
Cancer patients’ disease diagnosis status, treatment, and social network (%).

The mean age was 51 (SD = 14; range 22 to 89 years), 53 (SD = 13.9), and 49 (SD = 14.2) for all patients, patients with no distress, and socially distressed cancer patients, respectively. Figure 2 shows 50% of the cancer patients with uncontrolled disease status were socially distressed. However, there were more socially distressed patients with controlled disease status, diagnosed within a year, and undergoing treatment. More patients who shared their diagnosis with family, colleagues, and neighbors had no social distress.

Table 1 shows that distressed cancer patients were more common among men and younger than 50 years of age. Unlike patients who underwent surgery and received radiation therapy, those who received chemotherapy were more socially distressed.

Table 2 depicts the association of social distress and patients’ demographic profiles, disease status, treatment, and social networks, revealing that at the median (50th quantile), the odds of suffering from social distress are greater in patients 50 years or younger in age compared to older patients. However, at higher quantiles (e.g., 90% quantile), the odds of having social distress are lower for patients who underwent combined chemotherapy, surgery, and radiation therapies compared with patients without multiple treatments (OR = 0.65, CI, −0.820 to −0.036, *p* = 0.033). In addition, at the 95th quantile, the odds of suffering from social distress were 67% higher for patients diagnosed within one year than in patients diagnosed more than one year prior (OR = 1.664, CI, 0.100–0.918, *p* = 0.015). Strikingly, these factors differ considerably, having a strong effect on distress levels at higher quantiles.

The bounded logistic quantile regression (Table 3) suggests that sharing cancer diagnosis with social networks has a statistically significant moderating effect by attenuating the impact of disease status on social distress. There is a difference in the level of social distress between patients with uncontrolled disease condition and others based on social network. Patients with uncontrolled disease conditions but who have shared diagnosis and treatment with social networks have 48% lower social distress associated with cancer. We conducted post hoc correction tests to gauge whether there was a type 2 error. The tests confirmed that the non-significant variables did not have true effect on social distress.

## 4. Discussion

To adduce evidence in Saudi Arabia, this study examined social distress or toxicity and covariates in cancer patients, and how sharing diagnosis and treatment with social networks may attenuate the impact of disease status on social distress. We find that while more male patients were distressed, there were no statistically significant differences between genders. However, in a meta-analysis and narrative critical appraisal, women consistently reported more distress than men [32]. Women showed higher rates of anxiety and depression, which were more pronounced in some cancers [33].

Our findings suggest that cancer patients aged ≤50 years were significantly more likely to experience social distress due to cancer diagnosis and treatment compared with older patients. We find that this suggestion is consistent with literature suggesting that age-related differences in cancer-related suffering may be explained by treatment disparities. The way patients feel and cope with breast cancer, for example, can be affected by their age, how they are treated, and other things happening in their life. Evidence showed that younger patients were not only associated with severe psychological stress [34] but also complained of more physical and emotional stress symptoms than older patients [5]. Older women with breast cancer feel less sad, worried, and stressed than younger women who may have a harder time, needing more help to feel better [35,36,37]. A study that evaluated the QoL of younger and older women who had recently completed treatment for breast cancer discovered that younger women (<50 years) reported much more QoL disturbance, including emotional well-being and depression symptoms [36]. Younger patients may receive more intensive treatment, which may impair their QoL more than older patients. Because older breast cancer patients are more likely to have concomitant problems, younger women may receive more aggressive therapy, thereby jeopardizing their QoL [36,37].

This evidence of an age differential in QoL does not mean, however, that older patients cope with cancer-related stress all the time. A study in Saudi Arabia suggests that cancer is a sensitive topic and that most patients, especially the older ones, do not share and discuss their conditions [5]. Elsewhere, there is evidence that older people with cancer show fluctuations in psychological, physical, and social well-being over time, including a decline in depressive symptoms, physical performance, and role effectiveness [38]. Age-related impairments in various areas of the Comprehensive Geriatric Assessment (CGA) are associated with decreased health-related quality of life in older cancer patients [39].

In addition, our results suggest that the likelihood of suffering from social distress was significantly higher in patients diagnosed within one year than in patients diagnosed more than one year prior. This could be due to the shock of the diagnosis. Evidence suggests that newly diagnosed women, younger patients, and patients receiving combined therapies such as chemotherapy and radiation are more vulnerable to distress [22]. A previous cohort of patients from the same hospital in Saudi Arabia showed that patients diagnosed with cancer tended to have poorer emotional well-being, social function, and overall health in the first year after diagnosis [30].

Moreover, our analysis suggests that the likelihood of suffering from social distress was decreased in patients who underwent combined chemotherapy, surgery, and radiation therapies, a finding consistent with the literature. Evidence suggests that a treatment modality that combines two or more therapeutic agents is a foundation of cancer therapy [40]. Specifically, studies have shown that combination therapy not only is safe and well tolerated but also improves overall survival rate (6.4 months vs. 4 months) and progression-free survival compared with currently available therapeutics for this type of cancer [41,42]. Although they are associated with side effects and increased cost, in general, combined-modality therapies such as chemoradiation have demonstrated superior outcomes such as survival rates and better quality of life in various cancer types compared to a single therapy [43,44,45]. Evidence suggests that combinations of several classes of anticancer drugs/treatments have been shown to interfere with cancerous gene signaling, bone metastasis, and immune response known to be involved in the progression of prostate cancer [46,47]. Additionally, evidence reveals that ultrasound combined with microbubbles (USMB) in further combination with docetaxel produced a mean tumor inhibition of 73% [48].

In addition, our results suggest that sharing cancer diagnoses with social networks plays a moderating role by attenuating the impact of disease status on social burden. This implies that social networks were associated with higher QoL after a breast cancer diagnosis in a study that examined social networks and social support mechanisms [24]. There is also evidence suggesting that encouraging patients to share their experiences with cancer can help improve QoL, and that attachment security appears to promote social sharing [25]. There is evidence that the absence of close relationships and perceived sources of emotional support is significantly associated with increased breast cancer death rates, particularly among patients in the lowest quartile of reported close friends and relatives [26]. The literature also suggests that cancer screening increases when patients share their disease with social networks in the form of opinions from family, friends, and community members, particularly leaders [49,50]. This can help patients with proper diagnosis, effective timely treatment, identification of risk factors, and adaptation of this information to prevent or reduce the disease by modifying risk factors [51]. Elsewhere, less isolated patients and those who receive various types of social support, including sympathy or compassion, have been found to be associated with higher QoL for breast cancer survivors. [24,27].

### Contribution and Limitation

In Saudi Arabia, although there are studies on patient outcomes, particularly on predictors of QoL in cancer patients [5,28,29,30] our study provides new evidence that explores the broader association between social distress and covariates, including how the involvement of social networks and support may attenuate the impact of disease status on social distress. However, we recognize that our study is limited to a single large teaching and referral hospital. Therefore, there is a need for further research that isolates facility-specific cancer interventions as an experiment to assess the comparative practices and management styles of different facilities across the country.

## 5. Conclusions

Our investigation of the correlates of social distress in cancer patients found that men and patients younger than 50 years of age were more likely to experience stress. Social distress is more prevalent in patients diagnosed within a year and in controlled or uncontrolled situations. However, combining multiple treatment regimens appeared to have a positive effect on how patients perceived their social and economic situation. In addition, there is evidence that sharing cancer diagnoses with social networks attenuates the impact of disease status on social distress. Therefore, understanding the risk factors for social distress may be important for cancer management. Moreover, identifying the moderating role of sharing cancer diagnoses with social networks in mitigating the impact of disease status on social distress may provide valuable insights to healthcare providers in delivering holistic culture-specific care. Because treatment regimens that combine two or more therapies appear to be more beneficial for cancer patients, further investigation is needed. Although combined multiple treatments may result in superior outcomes such as survival rates for various cancer types compared to a single therapy, the associated side effects with intensive use of combined therapies should be evaluated considering the extent to which they affect QoL. Further, social networks in cancer facilities need to be promoted to reduce the burden associated with diagnosis and treatment. Not only will this complement and enable oncology professionals to provide better patient-centered care as part of an integrated approach, but it will also minimize the expected impact of initial shocks, as social distress appears to be more pronounced in patients diagnosed within a year and in controlled or uncontrolled situations. Because the effects of these risk factors are highly variable and have a strong impact on the magnitude of distress, at higher quantiles, patients who are more distressed need immediate attention. We acknowledge that our study was limited to a single large teaching and referral hospital. Thus, there is a need for further research that isolates facility-specific interventions as an experiment to assess the comparative practices and management styles of different cancer care management groups across the country.

## Figures and Tables

**Table 1:** Patients’ demographic and clinical characteristics.

Characteristic	Not Distressed, n (%)	Socially Distressed, n (%)	Total, n (%)
Mean social distress score; range	6.57 (SD = 4.07); Range: 1 to 19	−2.43 (SD = 3.74); Range: −18 to 1	1.84 (SD = 5.95); Range: −18 to 19
Distress level			
Not socially distressed			74 (47.4)
Socially distressed			82 (52.6)
Median age; range	53.5 (27–83)	49.1 (22–89)	51.2 (22–89)
Age category			
>50 years	42 (56.8)	33 (40.2)	75 (48.1)
≤50 years	32 (43.2)	49 (59.8)	81 (51.9)
Gender			
Males	27 (36.5)	37 (45.1)	64 (41.0)
Females	47 (63.5)	45 (54.9)	92 (59.0)
Metastatic			
No	51 (68.9)	63 (76.8)	114 (73.1)
Yes	23 (31.1)	19 (23.2)	42 (26.7)
Chemotherapy			
No	30 (40.5)	35 (42.7)	65 (41.7)
Yes	44 (59.5)	47 (57.3)	91 (58.3)
Surgery			
No	8 (10.8)	18 (21.9)	26 (16.7)
Yes	66 (89.2)	64 (78.1)	130 (83.3)
Radiation			
No	29 (39.2)	46 (56.1)	75 (48.1)
Yes	45(60.8)	36 (43.9)	81 (51.9)
Share diagnosis with family network			
No	18 (24.3)	25 (30.5)	43 (27.6)
Yes	56 (75.7)	57 (69.5)	113 (72.4)
Share diagnosis with colleagues and neighbors			
No	46 (62.2)	56(68.3)	102 (65.4)
Yes	28 (37.8)	26 (31.7)	54 (34.6)

**Table 2:** Logistic quantile regression of a bounded social distress score and associated factors.

Distress Score	Odds Ratio	95% CI		*p*-Value
Quantile 10				
≤50 years	1.157	−0.591	0.883	0.697
Gender = Male	0.864	−0.829	0.537	0.674
Metastatic = Yes	0.864	−0.737	0.445	0.627
Diagnosis within a year = Yes	0.887	−0.732	0.492	0.700
Chemo + Surgery + Radiation	0.681	−1.085	0.316	0.280
Disease present and uncontrolled = Yes	0.788	−0.975	0.498	0.523
Share with social network	0.999	−0.016	0.013	0.839
Intercept	0.638	−1.554	0.656	0.423
Quantile 25				
≤50 years	1.021	−0.362	0.403	0.915
Gender = Male	0.798	−0.562	0.111	0.187
Metastatic = Yes	0.718	−0.729	0.067	0.102
Diagnosis within a year = Yes	0.987	−0.332	0.306	0.934
Chemo + Surgery + Radiation	0.718	−0.692	0.029	0.071
Disease present and uncontrolled = Yes	0.815	−0.510	0.100	0.186
Share with social network	1.001	−0.004	0.006	0.684
Intercept	1.180	−0.471	0.802	0.608
Quantile 50				
≤50 years	1.235	0.000	0.422	0.049 *
Gender = Male	0.899	−0.311	0.099	0.309
Metastatic = Yes	0.899	−0.402	0.189	0.478
Diagnosis within a year = Yes	1.112	−0.120	0.333	0.356
Chemo + Surgery + Radiation	0.809	−0.454	0.031	0.087
Disease present and uncontrolled = Yes	0.900	−0.312	0.101	0.313
Share with social network	1.001	−0.002	0.004	0.520
Quantile 75				
≤50 years	1.238	−0.052	0.479	0.114
Gender = Male	0.901	−0.324	0.116	0.351
Metastatic = Yes	0.999	−0.382	0.379	0.994
Diagnosis within a year = Yes	1.111	−0.250	0.460	0.559
Chemo + Surgery + Radiation	0.810	−0.530	0.108	0.193
Disease present and uncontrolled = Yes	0.899	−0.398	0.184	0.470
Share with social network	1.001	−0.002	0.005	0.543
Intercept	1.300	−0.167	0.692	0.229
Quantile 90				
≤50 years	1.244	−0.125	0.561	0.211
Gender = Male	0.829	−0.532	0.158	0.285
Metastatic = Yes	0.921	−0.578	0.412	0.742
Diagnosis within a year = Yes	1.664	0.100	0.918	0.015 *
Chemo + Surgery + Radiation	0.652	−0.820	−0.036	0.033 *
Disease present and uncontrolled = Yes	1.002	−0.459	0.462	0.993
Share with social network	0.998	−0.006	0.003	0.400

* Significant at <5% level.

**Table 3:** Logistic quantile regression of a bounded social distress score and associated factors with disease status and social network interaction.

	Odds Ratio	95% CI		*p*-Value
Quantile 10				
≤50 years	1.293	−0.431	0.945	0.462
Gender = Male	0.864	−0.873	0.581	0.693
Metastatic = Yes	0.773	−0.910	0.396	0.438
Diagnosis within a year = Yes	1.000	−0.645	0.645	1.000
Chemo + Surgery + Radiation	0.675	−1.236	0.451	0.359
Disease present and uncontrolled = Yes	0.781	−1.020	0.526	0.529
Share with social network	0.999	−0.015	0.012	0.835
Disease # social network	1.147	−0.601	0.876	0.714
Quantile 25				
≤50 years	1.125	−0.235	0.471	0.510
Gender = Male	0.798	−0.546	0.094	0.166
Metastatic = Yes	0.718	−0.749	0.087	0.120
Diagnosis within a year = Yes	1.000	−0.356	0.356	1.000
Chemo + Surgery + Radiation	0.693	−0.769	0.035	0.073 **
Disease present and uncontrolled = Yes	0.910	−0.461	0.273	0.614
Share with social network	1.001	−0.004	0.006	0.683
Disease # social network	0.799	−0.727	0.279	0.379
Quantile 50				
≤50 years	1.171	−0.062	0.378	0.157
Gender = Male	0.900	−0.348	0.137	0.392
Metastatic = Yes	0.948	−0.401	0.294	0.760
Diagnosis within a year = Yes	1.171	−0.063	0.379	0.160
Chemo + Surgery + Radiation	0.809	−0.467	0.044	0.103
Disease present and uncontrolled = Yes	0.949	−0.352	0.247	0.728
Share with social network	1.001	−0.002	0.004	0.480
Disease # social network	0.854	−0.586	0.270	0.466
Quantile 75				
≤50 years	1.265	−0.022	0.492	0.072 **
Gender = Male	0.937	−0.342	0.212	0.643
Metastatic = Yes	0.937	−0.417	0.287	0.716
Diagnosis within a year = Yes	1.086	−0.230	0.396	0.601
Chemo + Surgery + Radiation	0.878	−0.448	0.188	0.421
Disease present and uncontrolled = Yes	1.048	−0.256	0.350	0.759
Share with social network	0.999	−0.004	0.002	0.603
Disease # social network	0.741	−0.726	0.128	0.168
Intercept	1.281	−0.231	0.726	0.308
Quantile 90				
≤50 years	1.446	−0.018	0.756	0.062 **
Gender = Male	1.000	−0.368	0.367	0.999
Metastatic = Yes	0.963	−0.530	0.454	0.879
Diagnosis within a year = Yes	1.319	−0.200	0.753	0.253
Chemo + Surgery + Radiation	0.605	−0.899	−0.107	0.013 *
Disease present and uncontrolled = Yes	1.112	−0.261	0.472	0.569
Share with social network	0.998	−0.007	0.002	0.325
Disease # social network	0.522	−1.165	−0.133	0.014 *

**#** Denotes disease status and social network interaction; * significant at <5% level; ** significant at <10% level.

## Data Availability

This study used previously collected data.
